# Detecting fitness epistasis in recently admixed populations with genome-wide data

**DOI:** 10.1186/s12864-020-06874-7

**Published:** 2020-07-11

**Authors:** Xumin Ni, Mengshi Zhou, Heming Wang, Karen Y. He, Uli Broeckel, Craig Hanis, Sharon Kardia, Susan Redline, Richard S. Cooper, Hua Tang, Xiaofeng Zhu

**Affiliations:** 1 Department of Mathematics, School of Science, Beijing Jiaotong University, Beijing, 100044 China; 2 Department of Population and Quantitative Health Sciences, Case Western Reserve University, Cleveland, OH 44106 USA; 3 Division of Sleep and Circadian Disorders, Brigham and Women’s Hospital and Harvard Medical School, Boston, MA USA; 4 Human and Molecular Genetics Center, Medical College of Wisconsin, Milwaukee, WI USA; 5 Department of Epidemiology, Human Genetics and Environmental Sciences, University of Texas Health Science Center at Houston, Houston, TX USA; 6 Department of Epidemiology, University of Michigan, Ann Arbor, MI USA; 7 Department of Public Health Science, Loyola University Medical Center, Maywood, IL USA; 8 Department of Genetics, Stanford University, Stanford, CA 94305 USA

**Keywords:** Fitness epistasis, Admixed population, Admixture linkage disequilibrium, Co-evolution, Diseases/traits

## Abstract

**Background:**

Fitness epistasis, the interaction effect of genes at different loci on fitness, makes an important contribution to adaptive evolution. Although fitness interaction evidence has been observed in model organisms, it is more difficult to detect and remains poorly understood in human populations as a result of limited statistical power and experimental constraints. Fitness epistasis is inferred from non-independence between unlinked loci. We previously observed ancestral block correlation between chromosomes 4 and 6 in African Americans. The same approach fails when examining ancestral blocks on the same chromosome due to the strong confounding effect observed in a recently admixed population.

**Results:**

We developed a novel approach to eliminate the bias caused by admixture linkage disequilibrium when searching for fitness epistasis on the same chromosome. We applied this approach in 16,252 unrelated African Americans and identified significant ancestral correlations in two pairs of genomic regions (*P*-value< 8.11 × 10^− 7^) on chromosomes 1 and 10. The ancestral correlations were not explained by population admixture. Historical African-European crossover events are reduced between pairs of epistatic regions. We observed multiple pairs of co-expressed genes shared by the two regions on each chromosome, including *ADAR* being co-expressed with *IFI44* in almost all tissues and *DARC* being co-expressed with *VCAM1, S1PR1* and *ELTD1* in multiple tissues in the Genotype-Tissue Expression (GTEx) data. Moreover, the co-expressed gene pairs are associated with the same diseases/traits in the GWAS Catalog, such as white blood cell count, blood pressure, lung function, inflammatory bowel disease and educational attainment.

**Conclusions:**

Our analyses revealed two instances of fitness epistasis on chromosomes 1 and 10, and the findings suggest a potential approach to improving our understanding of adaptive evolution.

## Background

Epistasis - defined as gene-gene interaction - has been found to play an important role in the etiology of complex diseases [1–3]. Epistasis is an important factor in shaping genetic variance within and between populations, and consequently phenotypic variation [1, 4–6]; epistasits is further considered to be one potential explanations of missing heritability in genome-wide association studies (GWAS) [7, 8]. Numerous statistical methods for detecting epistasis have been developed in recent years [9–11], including regression-based methods [12, 13], Bayesian statistical methods [14–16], linkage disequilibrium (LD)- and haplotype-based methods [17, 18] and machine-learning and data-mining methods [11, 19]. In general, the existing methods test for pairwise or higher-order interactions through either an exhaustive search of all marker combinations or a reduced marker set in the genome, which invariably lead to a large number of tests and reduced statistical power.

Fitness epistasis refers to the interactive effects among genetic variants at different loci on fitness, and has important consequences for adaptive evolution [20]. The genotype-fitness map, or the fitness landscape as introduced by Sewall Wright [21], is a visualization of a high-dimensional map, in which genotypes are organized in the x-y plane and fitness is plotted on the z axis [22]. The shape of the fitness landscape has been considered to have fundamental effects on the course of evolution [23]. Empirical information about the topography of real fitness landscapes has recently emerged from studies of mutations in the β-lactamase TEM1 [24], HIV-1 protease and reverse transcriptase [25] and *Drosophila melanogaster* recombinant inbred lines [26]. However, direct investigation of fitness epistasis in human subjects has thus far been limited [27–29]. Based on the assumption that functional interactive co-evolution could be maintained through complementary mutations over evolutionary history [27, 30], findings from a protein-protein network that used polygenetic distance metrics of a large-scale high-throughput protein-protein interaction dataset have suggested that Alzheimer’s disease (AD) associated genes, *PICALM, BIN1, CD2AP*, and *EPHA1* demonstrate evidence of a pattern of co-evolution [29]. A signature of co-evolution has also been observed for the killer immunoglobulin receptor (*KIR*) and the human leukocyte antigen *(HLA)* loci, where strong negative correlation exists between the gene frequencies of *KIR* and the corresponding *HLA* ligand [28]. Combinations of *KIR* and *HLA* variants have different degrees of resistance to infectious diseases that affect human survival during epidemics [31].

Fitness epistasis has the potential to generate linkage disequilibrium [32, 33] and affect the efficiency of natural selection [34, 35]. Similarly, we previously demonstrated that fitness epistasis can create LD among ancestry blocks in recently admixed populations such as African Americans and Hispanics, and this LD is detectable by testing the correlation of local ancestry between two unlinked loci [3]. Since ancestry blocks in recently admixed populations are often long and their frequencies are stable, testing the correlation between local ancestries is more powerful than testing the LD between single nucleotide polymorphisms (SNPs) in the genome by reducing the multiple comparison burden. Ancestry block LD can be generated as a result of population admixture, also termed admixture LD [36, 37]. It is then critical to separate the LD generated by fitness epistasis from admixture LD. To address this challenge, our previous study searched for fitness epistasis occurring on different chromosomes [3].

In this study, we developed a statistical approach to eliminate the bias caused by admixture LD when searching for fitness epistasis on the same chromosome. We applied the method in African Americans first by estimating the local ancestral correlation distribution under the null hypothesis that there is no fitness epistasis. Next, we searched for local ancestral correlations departing from the null distribution between two loci within each chromosome. To verify the identified fitness epistasis, we searched for pairs of tissue-specific co-expressed genes between the two identified regions on each chromosome by utilizing the GTEx V7 *cis*-eQTL expression dataset [38]. Finally, we examined whether there is an enrichment of diseases/traits associated with genes in the GWAS Catalog [39] within the fitness epistasis regions.

## Results

### Testing fitness epistasis on the same chromosome

**Fig. 1:**
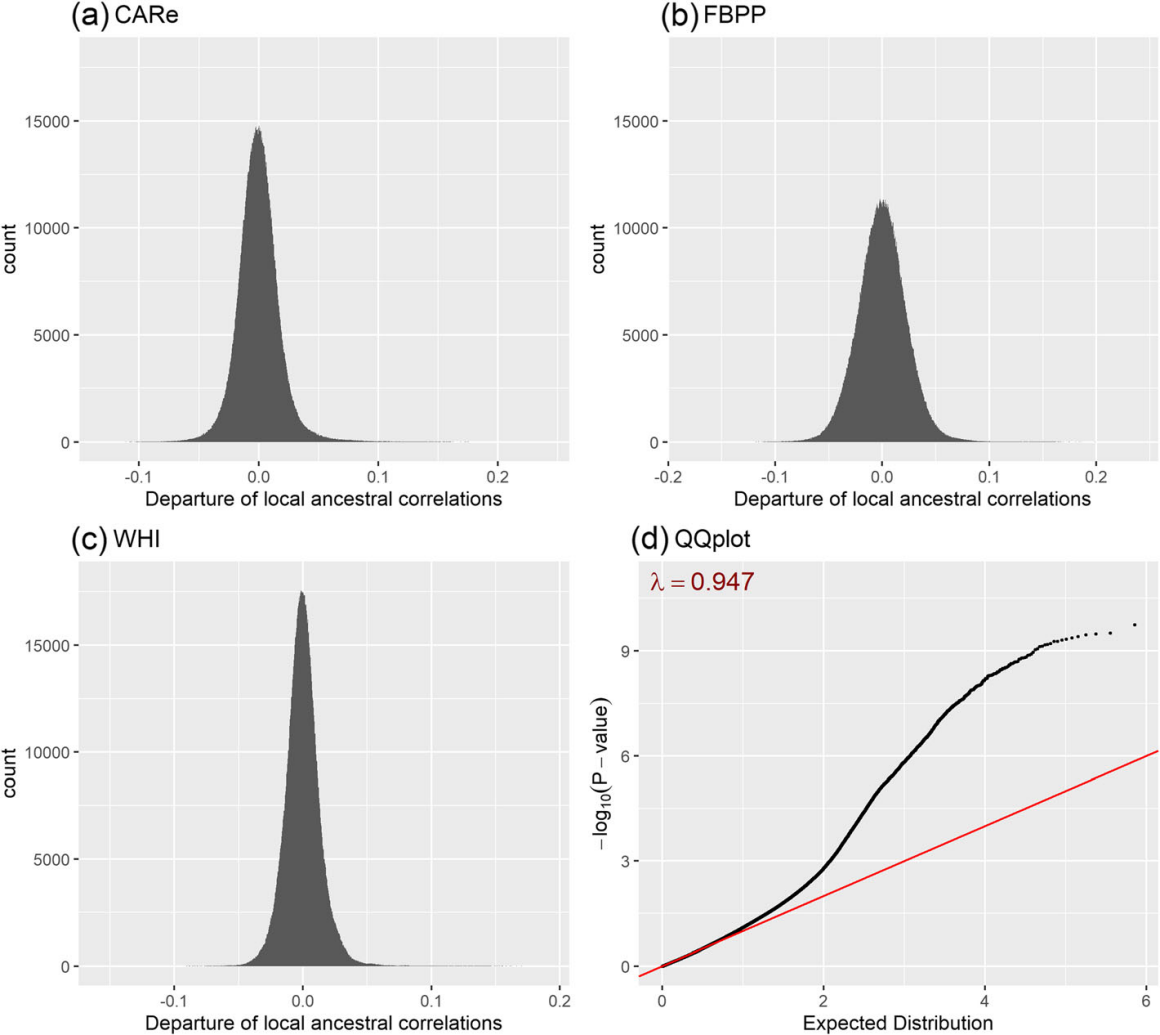
Distributions of the departure of local ancestral correlations and the corresponding statistical evidence. Distributions of the departure of local ancestral correlations in (**a**) CARe (sample size: 6238), (**b**) FBPP (sample size: 1864) and (**c**) WHI (sample size: 8150). (**d**) QQ-plot of *P*-values in meta-analysis (sample size: 16252)

We developed a novel statistical method to detect fitness epistasis on the same chromosome (see Materials and Methods). Our basic idea is that the ancestral correlations between two loci after eliminating the effect induced by population admixture suggests fitness epistasis [3]. We applied this method to the African Americans samples in the Candidate gene Association Resource (CARe), Family Blood Pressure Program (FBPP) and Women’s Health Initiative (WHI) cohorts. Our downstream analysis was based on 16,252 unrelated African Americans after removing related individuals and conducting quality controls (Table 1). The distributions of the departure of local ancestral correlations from the expected admixture LD on the same chromosomes are presented in Fig. 1a-c for the three datasets. We observed a significant departure from a normal distribution (the Kolmogorov–Smirnov test *p*-values < 2.2E-16). The skewness was 0.763, 0.245 and 0.925 for CARe, FBPP and WHI, respectively, suggesting the presence of fitness epistasis. The standard deviation of local ancestral correlations calculated between the pairwise loci located on different chromosomes in FBPP was larger than that of CARe and WHI, which can be attributed to the relatively small sample size of FBPP (Table 1). The QQ-plots of *P*-values for testing fitness epistasis for CARe, FBPP and WHI are presented in Figure S1. The genomic control parameter *λ* were all less than 1, suggesting our approach was conservative.

**Table 1:** Datasets, sample size and the standard deviation of correlations between pairwise loci on different chromosomes

	CARe	FBPP	WHI
Total sample size	8367	3636	8150
Unrelated sample size	6238	1864	8150
$\hat{\sigma}$^a^	0.015	0.027	0.012

^a^$\hat{\sigma}$ is the standard deviation of correlations between pairwise loci on different chromosomes

We conducted a fixed meta-analysis weighted by the square-root of the sample sizes to combine the results from the three cohorts [40]. The genomic control parameter *λ* in the meta-analysis was 0.947 (Fig. 1d). We observed multiple pairs of loci departing from the diagonal line, indicating fitness epistasis. We also performed Cochran’s *Q*-test to test the heterogeneity of locus pairs for the three cohorts. Among 1,440,130 locus pairs, 98.8% had *p*-values larger than 0.05, suggesting little heterogeneity. The pairwise correlations of Z-score among these three cohorts ranged from 0.241 to 0.411(Table S1), which were significantly larger than 0, suggesting shared fitness epistasis among the three cohorts.

**Fig. 2:**
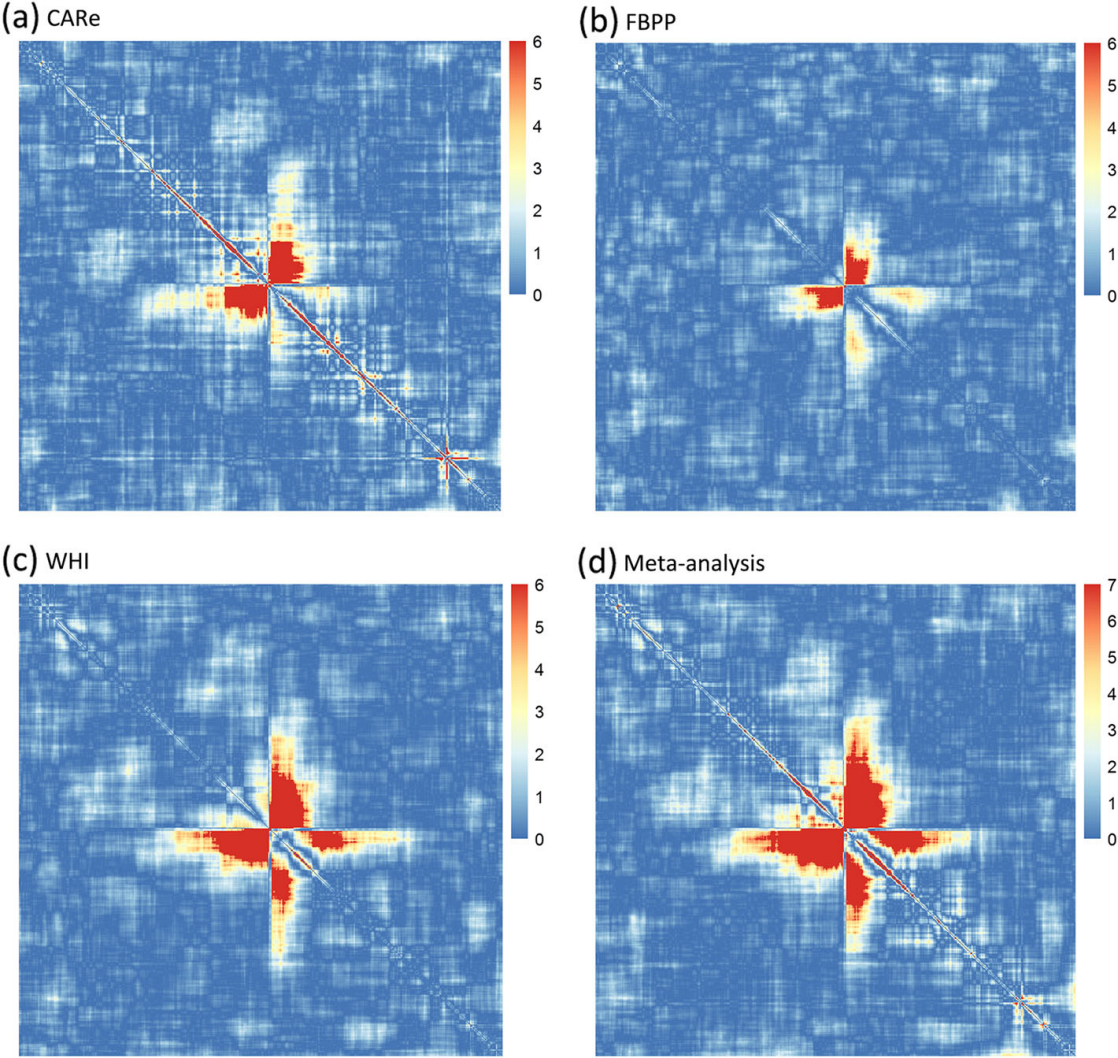
Heatmap of -log_10_(*P*-value) between pairwise loci located on chromosome 1 in (**a**) CARe, (**b**) FBPP, (**c**) WHI and (**d**) meta-analysis. Each point represents the -log_10_(*P*-value) between two loci. In (**a**), (**b**) and (**c**), if -log_10_(*P*-value) is larger than 6, we set the value as 6. In meta-analysis (**d**), if -log_10_(*P*-value) is larger than -log_10_(significant level), we set the value as 7, which reaches the significant level

**Fig. 3:**
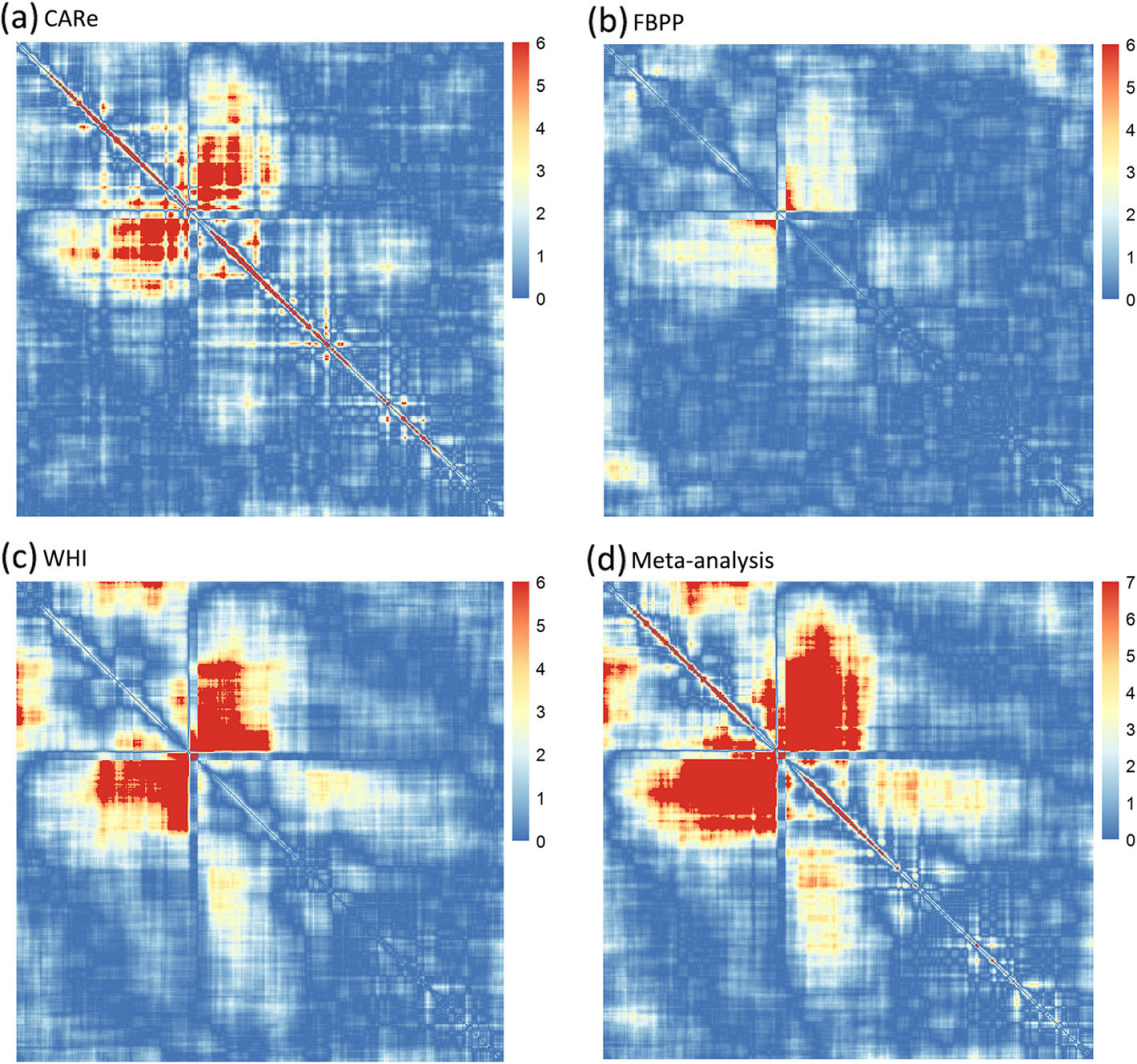
Heatmap of -log_10_(*P*-value) between pairwise loci located on chromosome 10 in (**a**) CARe, (**b**) FBPP, (**c**) WHI and (**d**) meta-analysis. Each point represents the -log_10_(*P*-value) between two loci. In (**a**), **b** and (**c**), if -log_10_(*P*-value) is larger than 6, we set the value as 6. In meta-analysis (**d**), if -log_10_(*P*-value) is larger than -log_10_(significant level), we set the value as 7, which reaches the significant level

There were 1,440,130 pairwise local ancestry correlation tests performed, and these correlations were dependent on the degree of admixture LD. We applied Bonferroni correction to adjust for the number of tests. We first calculated the number of independent bins (*k*_*i*_) for each chromosome *i* using the approach by Li and Jin [41]. The number of total independent tests in 22 chromosomes equals to ${\sum}_{i=1}^{22}\frac{k_i\left({k}_i-1\right)}{2}$. We estimated a total of 61,616 independent tests among 1,440,130 pairwise tests, yielding a significance level *α* = 8.11 × 10^− 7^. After excluding pairwise loci with a genetic distance less than 50 cM, we observed two pairs of genomic regions with significant evidence of fitness epistasis (*P*-value< 8.11 × 10^− 7^, Table 2). We did not observe any heterogeneity between these pairs of regions (all Cochran’s Q-test *p* values> 0.05). One pair of regions was localized to chr1:77.32–102.43 Mb and chr1:153.22–165.73 Mb and the other to chr10:10.26–24.59 Mb and chr10:55.20–73.20 Mb. The heatmaps of -log_10_(*P*-value) for pairwise loci on chromosomes 1 and 10 are presented in Figs. 2 and 3, respectively. On the heatmap of chromosome 1 (Fig. 2d), we observed two significant regions (red regions in Fig. 2). But the genetic distance between the pairwise loci in the region in the lower right quadrant was less than 50 cM; therefore, we excluded this signal due to the concern that admixture LD was not eliminated entirely. On the heatmap of chromosome 10, we also observed two significant regions in the meta-analysis (Fig. 3d). However, one of the red regions was near the telomere, which may reflect errors in local ancestry inference [42]. Therefore, this region was also excluded from further analyses. In the heatmaps of CARe, FBPP and WHI (Figs. 2a-c and 3 a-c), similar heatmap patterns were observed, suggesting that the fitness landscapes in CARe, FBPP and WHI were consistent.

**Table 2:** Significantly epistatic region pairs on the same chromosome

Chromosome	Region 1 (Mb)	Protein coding genes	Region 2 (Mb)	Protein coding genes
Chr 1	77.32–102.43	400	153.22–165.73	492
Chr 10	10.26–24.59	217	55.20–73.20	211

**Fig. 4:**
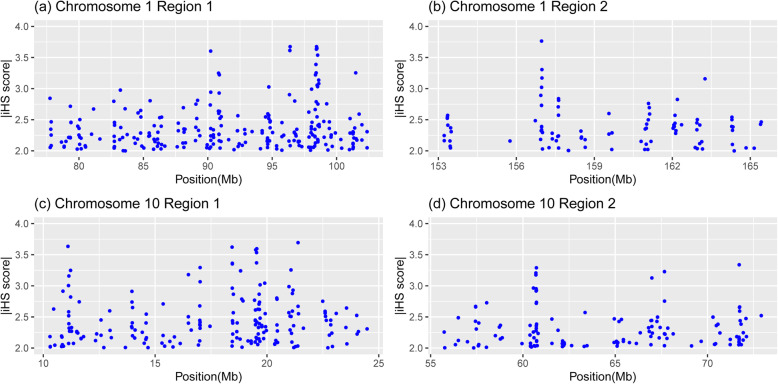
The recent selection signal (|iHS| > 2) on the epistatic regions in CARe cohort. (**a**) and (**b**) are the selection signal on region 1 (chr1:77.32–102.43 Mb) and region 2 (chr1:153.22–165.73 Mb) on chromosome 1, respectively. (**c**) and (**d**) are the selection signal on region 1 (chr10:10.26–24.59 Mb) and region 2 (chr10:55.20–73.20 Mb) on chromosome 10, respectively

We observed the largest proportion of African ancestry on chr1:153.22–165.73 Mb and the largest proportion of European ancestry on chr10:10.26–24.59 Mb (Figure S2). These two regions demonstrate substantial excess of local ancestry and may suggest natural selection. We calculated the integrated haplotype score (iHS) statistic [43] using *selscan* [44] in the four genomic regions using CARe samples (Fig. 4). We observed multiple loci with positive selection evidence (|iHS| > 2) in these four genomic regions. Similar signals could also be observed in ARIC, CARDIA, CFS, JHS and MESA cohorts separately (Figures S3 and S4).

If there were fitness epistasis between two loci on the same chromosome, then we would expect less recombination crossover events (or switch) between African and European chromosomes occurring between these two loci. We calculated the average number of crossovers between African and European chromosomes (ANCAEC) per centiMorgan in the region defined from the right boundary of region 1 and left boundary of region 2 (Table 2) on chromosomes 1 and 10 and then compared with the ANCAEC per centiMorgan in the rest of genome (Table S2). If fitness epistasis between two genomic regions was not present, then we would expect the ANCAEC per centiMorgan between the two regions to follow an approximately normal distribution, with the mean and variance estimated from the whole genome data after excluding the two regions. The ANCAEC per centiMorgan between the two detected regions on chromosome 1 is significantly less than what is present in the totality of the other domains in the genome (*P*-value = 7.51 × 10^− 35^), and similar results were observed on chromosome 10 (*P*-value = 2.53 × 10^− 7^), consistent with our findings of fitness epistasis in these two regions.

### Co-expression of genes in the two epistatic regions on chromosome 1 and 10

We hypothesized that the regions demonstrating fitness epistasis will likely harbor co-expressed genes in multiple tissues, attributable to genes of similar function. We identified genes residing within the four regions on chromosomes 1 and 10 using the GENCODE dataset [45]. In these four regions there are known to reside 400, 492, 217 and 211 protein-coding genes (chr1:77.32–102.43 Mb; chr1:153.22–165.73 Mb; chr10:10.26–24.59 Mb and chr10:55.20–73.20 Mb), respectively. GTEx V7 tissue-specific normalized gene expression matrices and covariates were downloaded from the GTEx Portal (https://www.gtexportal.org/home/datasets). We calculated residuals of gene expression after adjusting for sex, platform, the first three principal components and tissue-specific latent factors inferred by the GTEx consortium using the PEER method [46]. We performed pairwise gene expression correlation analysis using the residuals of gene expression between genes in regions 1 and 2 of chromosome 1. Similar analysis was performed for the gene pairs between genes in regions 1 and 2 of chromosome 10. We applied Bonferroni correction to adjust for the number of tests, which was calculated by the number of independent genes in region 1 multiplied by the number of independent genes in region 2 for a pair of epistatic regions. We calculated the number of independent genes in a region using the approach by Li and Jin [41]. For each tissue, the number of genes expressed in each region varies, but we used the maximum number of independent genes when adjusting for multiple comparisons. Our calculations established the significance levels of 1.689 × 10^− 6^ and 5.261 × 10^− 6^ for chromosomes 1 and 10, respectively. Because gene expressions are correlated across tissues [47], we did not correct for the number of tissues. The thresholds we used adopted to a false discovery rate of < 5% for both chromosome 1 and 10.

**Fig. 5:**
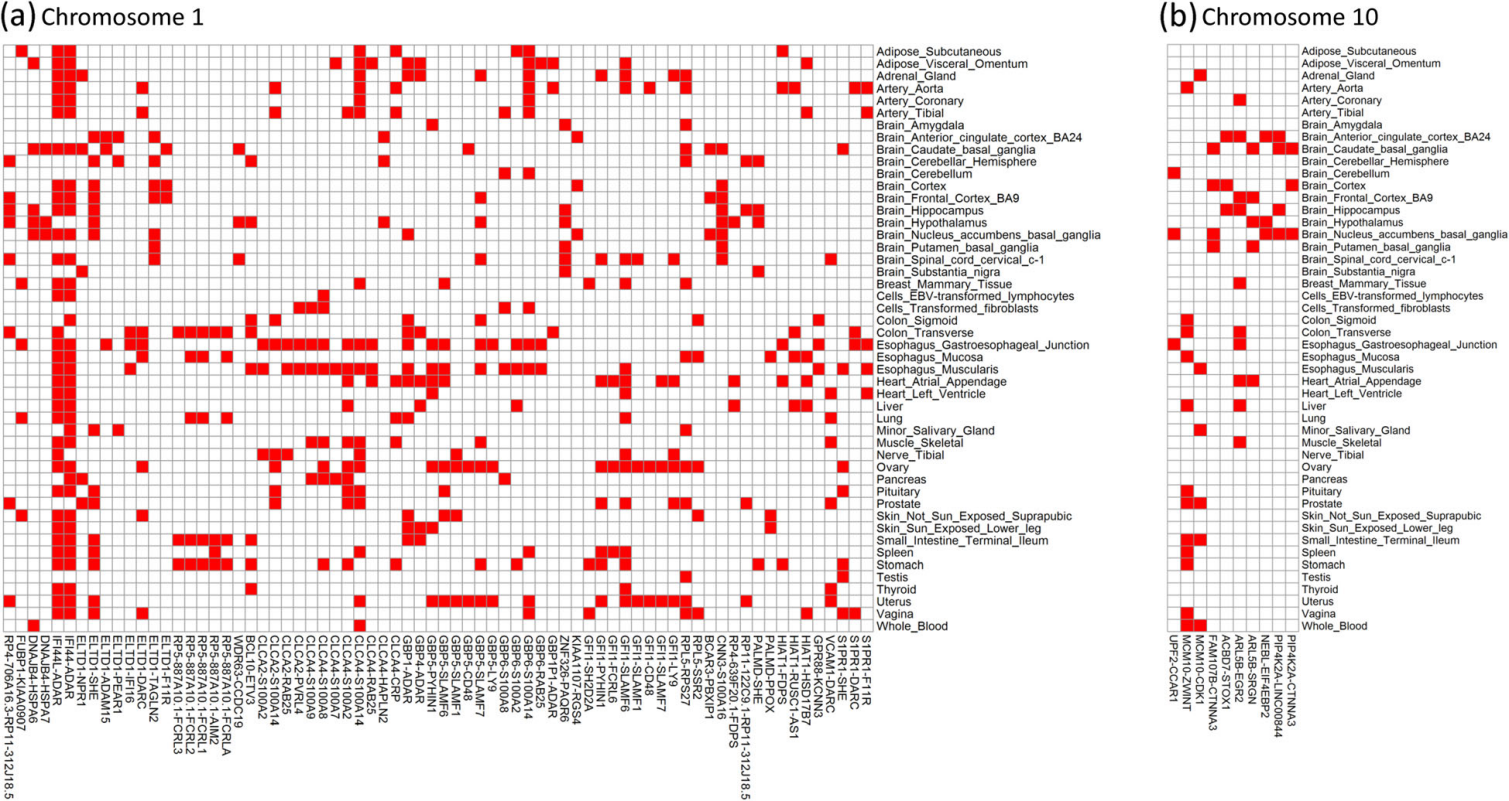
Heatmap of *P*-values of significantly co-expressed gene pairs located on (**a**) chromosome 1 and (**b**) chromosome 10 in different tissues. Y-axis represents the names of different tissues. X-axis represents the names of gene pairs. These gene pairs are significantly co-expressed in more than 2 tissues. Red block represents the significant signals

We observed 599 pairs of genes that are significantly co-expressed in the epistatic regions on chromosome 1, and 161 pairs of genes that are co-expressed in the epistatic regions on chromosome 10, for at least 1 tissue. We performed a tissue-specific enrichment analysis for these co-expressed genes with the GENE2FUNC option implemented in FUMA [48]. Across 53 tissue types, an enrichment test of differentially expressed genes (DEG) showed significantly higher co-expression of these genes in the lung (*P*-value < 0.05/53) (Figure S5). The heatmaps of the -log_10_(*P*-value) for these co-expressed gene pairs on chromosomes 1 and 10 are shown in Figures S6-S7, respectively. We observed multiple significantly co-expressed gene pairs in multiple tissues (Fig. 5). For example, *IFI44* and *ADAR* are co-expressed in almost all tissues in the GTEx data. We also observed the *DARC* gene, which encodes the Duffy antigen receptor for human malaria [49], was significantly co-expressed with *VCAM1, S1PR1* and *ELTD1* in multiple tissues. The proportion of significant co-expressed gene pairs in epistatic regions was substantially higher than the regions that did not overlap with the epistatic regions on chromosome 1 and chromosome 10 (Table S3).

### Enrichment of diseases/traits-associated genes from the GWAS catalog in epistatic regions

GWAS have identified genetic variants that are significantly associated with phenotypes, typically in large sample cohorts. We hypothesized that GWAS hits for the co-expressed gene pairs may have the same disease/phenotype. We compared the GWAS hits on the epistatic regions with the remaining regions by examining the genome wide signals from the GWAS Catalog [39]. We observed an approximate 2-fold enrichment in region 2 of chromosome 1 compared with the average number of GWAS hits on chromosome 1 (Table S4). To calculate the *P*-value of the enrichment, we divided the chromosomes into non-overlapping regions after excluding the target region and then calculated the average number of hits and the corresponding standard error. The *P*-value of the enrichment was calculated by a Z-score, which was defined as the difference between the observed number of GWAS hits in a target region and the average number of GWAS hits, divided by the standard error. We assumed that the Z-score followed a standard normal distribution. The enrichment in region 2 of chromosome 1 was statistically significant (*P*-value = 0.0099, Table S4), suggesting that the epistatic region likely harbors more GWAS hits. We also observed 15 pairs of genes associated with the same diseases/traits on chromosomes 1 and 10 (Table 3). Among them, 5 pairs of genes have GWAS hits for multiple traits.

**Table 3:** Co-expressed gene pairs and their common associated diseases/traits

Chr	Gene in region 1	Gene in region 2	Disease/trait	Significant tissues
Chr 1	AK5	CADM3	Educational attainment (years of education)	Brain Nucleus accumbens basal ganglia
Chr 1	DPH5	DAP3	Inflammatory bowel disease	Uterus
Chr 1	ELTD1	DARC	White blood cell count	Artery Aorta;Artery Tibial; Colon Transverse; Esophagus Gastroesophageal Junction; Esophagus Mucosa; Ovary; Skin Not Sun Exposed Suprapubic; Vagina
Chr 1	ELTD1	DCST2	Eosinophil counts	Adipose Subcutaneous
Chr 1	GFI1	SLAMF7	Multiple sclerosis	Heart Atrial Appendage; Ovary; Uterus
Chr 1	MIR137HG	CADM3	Educational attainment (years of education)	Brain Hypothalamus
Chr 1	MTF2	NDUFS2	Eosinophil counts	Brain Hippocampus
Chr 1	PKN2	ASH1L	Red blood cell count	Skin Not Sun Exposed Suprapubic
Chr 10	CAMK1D	JMJD1C	General cognitive ability	Brain Hypothalamus
Chr 10	CAMK1D	JMJD1C	Educational attainment	Brain Hypothalamus
Chr 10	CAMK1D	JMJD1C	Educational attainment (years of education)	Brain Hypothalamus
Chr 10	CAMK1D	JMJD1C	Highest math class taken	Brain Hypothalamus
Chr 10	CAMK1D	JMJD1C	Lung function (FEV1/FVC)	Brain Hypothalamus
Chr 10	CAMK1D	JMJD1C	Educational attainment	Brain Hypothalamus
Chr 10	CELF2	CCDC6	Systolic blood pressure	Spleen
Chr 10	CELF2	CCDC6	Pulse pressure	Spleen
Chr 10	FAM107B	CTNNA3	Night sleep phenotypes	Brain Caudate basal ganglia; Brain Cortex; Brain Nucleus accumbens basal ganglia; Brain Putamen basal ganglia
Chr 10	FRMD4A	REEP3	Red blood cell count	Breast Mammary Tissue
Chr 10	NEBL	JMJD1C	Interleukin-10 levels	Pituitary
Chr 10	NEBL	JMJD1C	Lung function (FEV1/FVC)	Pituitary
Chr 10	PIP4K2A	CTNNA3	Breast cancer	Brain Caudate basal ganglia; Brain Cortex; Brain Nucleus accumbens basal ganglia
Chr 10	PIP4K2A	CTNNA3	Obesity-related traits	Brain Caudate basal ganglia; Brain Cortex; Brain Nucleus accumbens basal ganglia
Chr 10	PLXDC2	BICC1	Heel bone mineral density	Stomach
Chr 10	PLXDC2	BICC1	Pulse pressure	Stomach

## Discussion

In this study, we developed a novel statistical method to detect fitness epistasis by testing the correlation between local ancestries on the same chromosome in a recently admixed population while eliminating potential bias caused by admixture LD. Applying our method to three large African American cohorts, CARe, FBPP and WHI, we identified two significant epistatic genomic region pairs on chromosomes 1 and 10. These genomic regions also demonstrated high iHS scores, suggesting signatures of natural selection. We observed that historical recombination events are less likely to occur between a pair of epistatic genomic regions. A large number of gene pairs on the chromosomes 1 and 10 epistatic regions are co-expressed in multiple tissues in the GTEx data. Furthermore, multiple co-expressed gene pairs in these epistatic regions are associated with the same diseases/traits in the GWAS Catalog.

Several statistical methods for detecting epistasis have been developed, either by exhaustively testing all possible pairwise interactions between SNPs or performing similar tests in a reduced SNP set. The pairwise searching methods that use genotyping array data would require billions of pairwise tests, which are computationally inefficient and result in a high statistical penalty because of the multiple testing burden [9]. In our method, we tested pairwise interactions between the ancestral blocks on the same chromosome in a recently admixed population. The current approach can be viewed as an extension of our previous study [3], which focused on pairs of ancestries on different chromosomes. This approach is more powerful because the ancestral blocks are long and often extend beyond 50 cM [36, 50]. We divided the genome into 400 kb bins and used the middle marker of each bin to represent the local ancestries of the corresponding bins [3]. This is reasonable because of the long admixture LD. It is well known that the local ancestries in neighboring bins are highly correlated. Therefore, we applied the widely used method by Li and Jin [41] to calculate the number of independent tests to determine the significance level. Our method could still be conservative because the genomic control values in CARe, FBPP and WHI - as well as in the meta-analysis - were all less than 1. We observed two significant epistatic regions on chromosomes 1 and 10 in the meta-analysis. The general correlation patterns were similar across the stratified analysis in CARe, FBPP and WHI cohorts (Figs. 2 and 3), suggesting that the detection of fitness epistasis regions was not likely due to chance. We also observed that the pairwise correlations of Z-scores among these three cohorts ranged from 0.241 to 0.411(Table S1). These significant correlations suggested that there was shared fitness epistasis among the three cohorts. If there were no fitness epistasis between a pair of regions, the Z-scores from different cohorts would be independent and the correlation should be close to 0. It is possible that population admixture could have lead to correlations of Z-scores among the three cohorts. However, we carefully modeled and excluded the contribution by the population admixture (see Materials and Methods). The genomic control parameters of the QQ plots of the Z-scores were all under 1, suggesting that population admixture was well controlled.

The gene pairs that likely contribute to the detected fitness epistasis are co-expressed in multiple tissues and associated with the same traits on the epistatic regions on chromosome 1 and 10. *ELTD1* and *DARC* are co-expressed in multiple tissues (Table 3 and Fig. 5) and also associated with white blood cell count [51, 52]. Both *ELTD1* and *DARC* have been reported to be under selection pressure [53, 54]. *DARC* encodes the Duffy antigen receptor for human malarial parasites and *ELTD1* plays an essential role in heart development and the prevention of cardiac hypertrophy. The genes *DPH5* and *DAP3* are co-expressed and associated with inflammatory bowel disease (IBD) [55]. IBD is a chronic inflammatory and autoimmune disease that plays an important role in pathogen defense and other functions that are under strong natural selection in humans; thus, the associated genes will exert a negative influence on reproductive fitness [56]. Gene pairs *ELTD1-DCST2* and *MTF2*-*NDUFS2* are associated with eosinophil counts [51]. Gene pairs *PKN2*-*ASH1L* and *FRMD4A*-*REEP3* are associated with red blood cell count [51]. Variation in red and white blood cell count are associated with allergic diseases and certain infections [52, 57, 58], which play important roles in natural selection. We also observed several gene pairs associated with educational attainment, such as gene pairs *AK5*-*CADM3*, *MIR137HG*-*CADM3,* and *CAMK1D*-*JMJD1C* [59, 60]. These gene pairs are all co-expressed in brain tissues (see Table 3) and involved in brain-development processes and neuron-to-neuron communication [59]. Two recent studies suggest on-going negative selection against education attainment in Western European populations [61, 62]. Other interesting gene pairs associated with the same diseases/traits are shown in Table 3. We note that *IFI44* and *ADAR* are co-expressed in almost all the tissues in the GTEx data (Fig. 5). It has been reported that *IFI44* is associated with psychiatric disorders [63], febrile seizures [64], immune response to measles vaccine (measles-specific neutralising antibody titre) [65] and asthma [66], and *ADAR* is associated with Aicardi–Goutières syndrome [67], cerebrospinal fluid levels of Alzheimer’s disease-related proteins [68], lung cancer [69] and prostate cancer [70]. Psychiatric disorders are moderately to highly heritable and also highly disabling and confer decreasing fitness as observed in schizophrenia [71]. A recent study also suggested that genetic variations associated with Alzheimer’s disease and asthma were less common in people who lived longer [72].

As mentioned above, most of the diseases/traits listed in Table 3 have genetic evidence for natural selection in humans, although this would reflect the marginal effect of a single gene. Fitness epistasis leaves genomic signatures as a result of co-evolution through a trait. One way that a gene may modify a trait is by affecting gene regulation in different tissues. This may be a mechanism that explains fitness epistasis for co-expressed genes. Thus, our study adds evidence to the hypothesis that genetic interactions contribute to human fitness, a phenomenon incompletely explored in prior literature.

Using the enrichment of GWAS hits to strength our finding of fitness epistasis is a potential limitation inherent in this analysis. In the GWAS Catalog, the associated genetic variants were mapped based on the gene and variant positions. A significant variant from GWAS may actually regulate a gene far away from the variant. Therefore, our analysis based on gene and variant positions may not truly reflect the GWAS hit enrichment and the current enrichment estimation may be conservative.

It is worth noting that our approach is only applicable to recently admixed populations such as African Americans or Hispanics. One of our proposed future directions to extend this method would involve more complex admixed populations, such as the Uygur and Tibetan populations. In addition, the efficiency of our method is influenced by the accuracy of the local ancestry inference. With additional whole genome sequencing data becoming readily available, inference of local ancestry can be improved. We expect more genomic regions with fitness epistasis will be identified in the near future.

## Conclusions

In summary, detecting fitness epistasis is extremely challenging, especially in human populations. Our method takes advantage of a recently admixed population and reliable local ancestry inference using genetic variants from genotyping array data. The potential contribution of this approach is supported by the analysis using empirical data. Our analyses revealed two instances of fitness epistasis on chromosomes 1 and 10, and the findings provide novel insight into our understanding of adaptive evolution.

## Materials and methods

### Admixture LD in an admixed population

In the hybrid isolation model, the admixture LD (*D*) decay between two loci without epistasis can be approximated by an exponential function [73, 74],

$$ D={D}_0{\left(1-d\right)}^t\approx {D}_0{e}^{- td}, $$

where *d* is the genetic distance between the two loci and *t* is the time elapsed since the initial admixture event (admixture time). Admixture LD decay is more complex in the continuous gene flow model [36]. However, this exponential function can well mimic our data, as demonstrated in the WHI African American samples (Figure S8). We observed that this exponential function well fits the empirical admixture LD curve. We did observe that there were departures from the fitting line, especially with distance over 50 cM, which may be attributed to statistical noise or fitness epistasis. Our goal is to separate fitness epistasis from the statistical noise.

### Estimate the departure from the admixture LD curve

Let *X*_*i*_ be the local ancestry at locus *i* and *X*_*j*_ be the local ancestry at locus *j*. We assumed the two loci are located on the same chromosome. We denoted *β*_*ij*_ as the observed correlation of local ancestries between loci *i* and *j,*

$$ {\beta}_{ij}= corr\left({X}_i,{X}_j\right) $$

Let *f*(*d*) be an exponential function representing the admixture LD between two loci with genetic distance (*d*) under no fitness epistasis,

$$ f(d)={a}_0+{a}_1\exp \left(-{a}_2d\right), $$

where ***a =*** (*a*_0_, *a*_1_, *a*_2_) is the vector of parameters in the exponential function. We added a parameter *a*_0_, which represents a background LD when the two loci are unlinked.

For each locus *i*, we calculated the correlation of local ancestries *β*_*ij*_ between loci *i* and *j* for all *j* ≠ *i* on the same chromosome using genotyping array data [3]. We fit a nonlinear regression model by optimizing the following function,

$$ {\hat{\boldsymbol{a}}}^{\boldsymbol{i}}=\underset{\boldsymbol{a}}{\mathrm{argmin}}\left(\sum \limits_{j\ne i}{\left({\beta}_{ij}-f\left({d}_{ij}\right)\right)}^2\right). $$

We predicted the admixture LD between loci *i* and *j* under the null of no fitness epistasis by

$$ {\hat{\beta}}_{ij}={\hat{a}}_0^i+{\hat{a}}_1^i\exp \left(-{\hat{a}}_2^i{d}_{ij}\right). $$

The departure of observed admixture LD from the expected admixture LD is calculated by

$$ {\left({\beta}_{res}\right)}_{ij}={\beta}_{ij}-{\hat{\beta}}_{ij}. $$

The above calculation can also be applied to estimate ${\hat{\beta}}_{ji}$, that is, given locus *j,* we can estimate ${\hat{\boldsymbol{a}}}^{\boldsymbol{j}}$ and therefore ${\hat{\beta}}_{ji}$ and (*β*_*res*_)_*ji*_. In theory, ${\hat{\beta}}_{ij}={\hat{\beta}}_{ji}$. But slight variation can be observed because different pairwise loci are applied. Thus, we averaged (*β*_*res*_)_*ij*_ and (*β*_*res*_)_*ji*_ as the final departure of observed admixture LD from the expected admixture LD when no fitness is present,

$$ {\left({\hat{\beta}}_{res}\right)}_{ij}=\frac{{\left({\beta}_{res}\right)}_{ij}+{\left({\beta}_{res}\right)}_{ji}}{2}. $$

### Testing for fitness epistasis

When there is no fitness epistasis, the departure (${\hat{\beta}}_{res}$) of admixture LD from the null follows a normal distribution $\left.{\hat{\beta}}_{res}\sim N\Big(0,{\sigma}^2\right)$, where *σ*^2^ is the unknown variance. This variance can be estimated by the local ancestral correlations between two loci on different chromosomes, as suggested by Wang et al. [3]. Since the genetic distance between two loci located on different chromosomes was expected to be infinite, the standard deviation of local ancestral correlations between these loci was therefore served as the population standard deviation of the local ancestral correlations. Thus, we estimated *σ* by using the standard deviation of ancestral correlations among the loci located on the different chromosomes. To test fitness epistasis, we applied a Z-test ${Z}_{ij}=\frac{{\left({\hat{\beta}}_{res}\right)}_{ij}}{\hat{\boldsymbol{\sigma}}}$, with the *P*-value calculated by *P*_*ij*_ = 2(1 − *ϕ*(|*Z*_*ij*_|)).

### Dataset

We applied our method to three African American cohorts: (1) the CARe study initiated by National Heart, Lung, and Blood Institute, which included 8367 individuals from five studies: the Atherosclerosis Risk in Communities study (ARIC), the Coronary Artery Risk Development in Young Adults study (CARDIA), the Cleveland Family Study (CFS), the Jackson Heart Study (JHS), and the Multi-Ethnic Study of Atherosclerosis (MESA) [75]. All the samples were genotyped using the Affymetrix 6.0 platform. These genotype data were downloaded from the dbGAP repository: ARIC: dbGaP phs000280.v1.p1, CARDIA: dbGaP phs000285.v2.p2, CFS: dbGaP phs000284.v2.p1, JHS: dbGaP phs000499.v4.p2, MESA: dbGaP phs000283.v7.p3. (2) the NHLBI Family Blood Pressure Program (FBPP), which collected 3636 African American subjects from three networks, GenNet, GENOA (dbGaP phs000379.v1.p1), and HyperGEN (dbGaP phs001293.v2.p1) [76], who were genotyped using either Affymetrix 6.0 or Illumina 1 M platform; (3) the Women’s Health Initiative (WHI), which includes 8150 African American postmenopausal women, who were genotyped with the Affymetrix 6.0 platform (dbGaP phs000386.v8.p3). QCs were described in Wang et al. (2017) [3]. We excluded related samples and samples with extremely low (≤ 5%) or high (≥ 98%) African proportions. Our downstream analysis was based on 16,252 unrelated African Americans after quality control (Table 1).

We first inferred the local ancestries for the three cohorts with SABER+ [77]. SABER+ was designed to reconstruct genetic ancestral blocks in admixed populations based on the Markov-hidden Markov model. Following the analysis procedure of our previous study [3], we divided the genome into 7389 bins with an average length of 400 kb due to high correlations between adjacent local ancestries. The local ancestry at the middle marker was used to represent the local ancestry of each bin. Due to potentially high local ancestry inference errors on the telomeres and centromeres [42], we excluded bins located within 2 Mb of these two types of regions from the analysis. We further performed meta-analysis to combine the results from the three datasets using the weighted Z-score method as described in the METAL software [40]. Finally, we used gene expression data from the GTEx dataset [38] and diseases/traits associations from the GWAS Catalog [39] to strengthen our findings of fitness epistasis.

## Supplementary information

**Additional file 1:Figure S1.** QQ-plot of *P*-values in (a) CARe, (b) FBPP and (c) WHI cohort. **Figure S2.** Proportion of local ancestries of (a) chromosome 1 and (b) chromosome 10. **Figure S3.** The recent selection signal (|iHS| > 2) on the epistatic regions on chromosome 1 in CARe cohort. **Figure S4.** The recent selection signal (|iHS| > 2) on the epistatic regions on chromosome 10 in CARe cohort. **Figure S5.** The tissue expression results of co-expressed genes on 53 tissue types by GTEx in FUMA. **Figure S6.** Heatmap of *P*-value of significantly co-expressed gene pairs located on epistatic regions on chromosome 1 in different tissues. **Figure S7.** Heatmap of *P*-value of significantly co-expressed gene pairs located on epistatic regions on chromosome 10 in different tissues. **Figure S8.** An example of admixture LD decay with genetic distance. **Table S1.** Correlations of Zscore among CARe, FBPP and WHI cohorts. **Table S2.** Number of crossover events between African and European chromosomes**. Table S3.** Comparison of co-expressed gene pairs between the epistatic regions and other regions not overlapped with the epistatic regions on chromosome 1 and chromosome 10. **Table S4.** Number of GWAS hits on the epistatic regions.

## Data Availability

All data are available on dbGaP: ncbi.nlm.nih.gov/gap ARIC: phs000280.v1.p1; CARDIA: phs000285.v2.p2; CFS: phs000284.v2.p1; JHS: phs000499.v4.p2; MESA: phs000283.v7.p3; GENOA: phs000379.v1.p1; HyperGEN: phs001293.v2.p1; WHI: phs000386.v8.p3. GTEx: https://www.gtexportal.org/home/datasets. The V7 data is under the name: GTEx Analysis V7 (dbGaP Accession phs000424.v7.p2).

## Abbreviations

AD: Alzheimer’s disease
ANCAEC: Average number of crossovers between African and European chromosomes
ARIC: The Atherosclerosis Risk in Communities study
CARDIA: The Coronary Artery Risk Development in Young Adults study
CARe: Candidate gene Association Resource
CFS: The Cleveland Family Study
DEG: Differentially expressed genes
FBPP: The Family Blood Pressure Program
GTEx: The Genotype-Tissue Expression
GWAS: Genome-wide association studies
HLA: The human leukocyte antigen
IBD: Inflammatory bowel disease
iHS: Integrated haplotype score
JHS: The Jackson Heart Study
KIR: Killer immunoglobulin receptor
LD: Linkage disequilibrium
MESA: The Multi-Ethnic Study of Atherosclerosis
NHLBI: National Heart, Lung, and Blood Institute
SNPs: Single nucleotide polymorphisms
WHI: Women’s Health Initiative
