# Supplementary material for: Detecting fitness epistasis in recently admixed populations with genome-wide data
Source: BMC Genomics. 2020 Jul 11;21:476. doi: 10.1186/s12864-020-06874-7 (PMC7353720; doi:10.1186/s12864-020-06874-7)
Supplement: Supplementary file 1 — Additional file 1:Figure S1. QQ-plot of P-values in (a) CARe, (b) FBPP and (c) WHI cohort. Figure S2. Proportion of local ancestries of (a) chromosome 1 and (b) chromosome 10. Figure S3. The recent selection signal (|iHS| > 2) on the epistatic regions on chromosome 1 in CARe cohort. Figure S4. The recent selection signal (|iHS| > 2) on the epistatic regions on chromosome 10 in CARe cohort. Figure S5. The tissue expression results of co-expressed genes on 53 tissue types by GTEx in FUMA. Figure S6. Heatmap of P-value of significantly co-expressed gene pairs located on epistatic regions on chromosome 1 in different tissues. Figure S7. Heatmap of P-value of significantly co-expressed gene pairs located on epistatic regions on chromosome 10 in different tissues. Figure S8. An example of admixture LD decay with genetic distance. Table S1. Correlations of Zscore among CARe, FBPP and WHI cohorts. Table S2. Number of crossover events between African and European chromosomes. Table S3. Comparison of co-expressed gene pairs between the epistatic regions and other regions not overlapped with the epistatic regions on chromosome 1 and chromosome 10. Table S4. Number of GWAS hits on the epistatic regions. [file 12864_2020_6874_MOESM1_ESM.docx]

**Supplementary Figures**


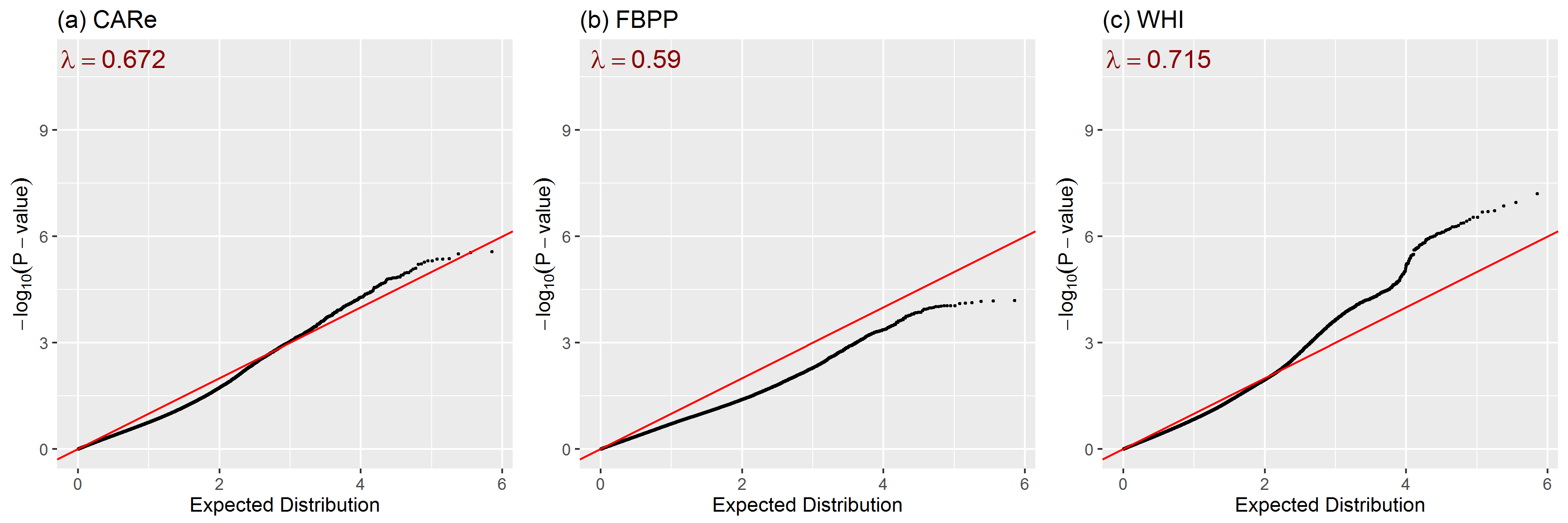


**Figure S1. QQ-plot of P-values in (a) CARe, (b) FBPP and (c) WHI cohort.** $\lambda$ is the genomic control parameter.


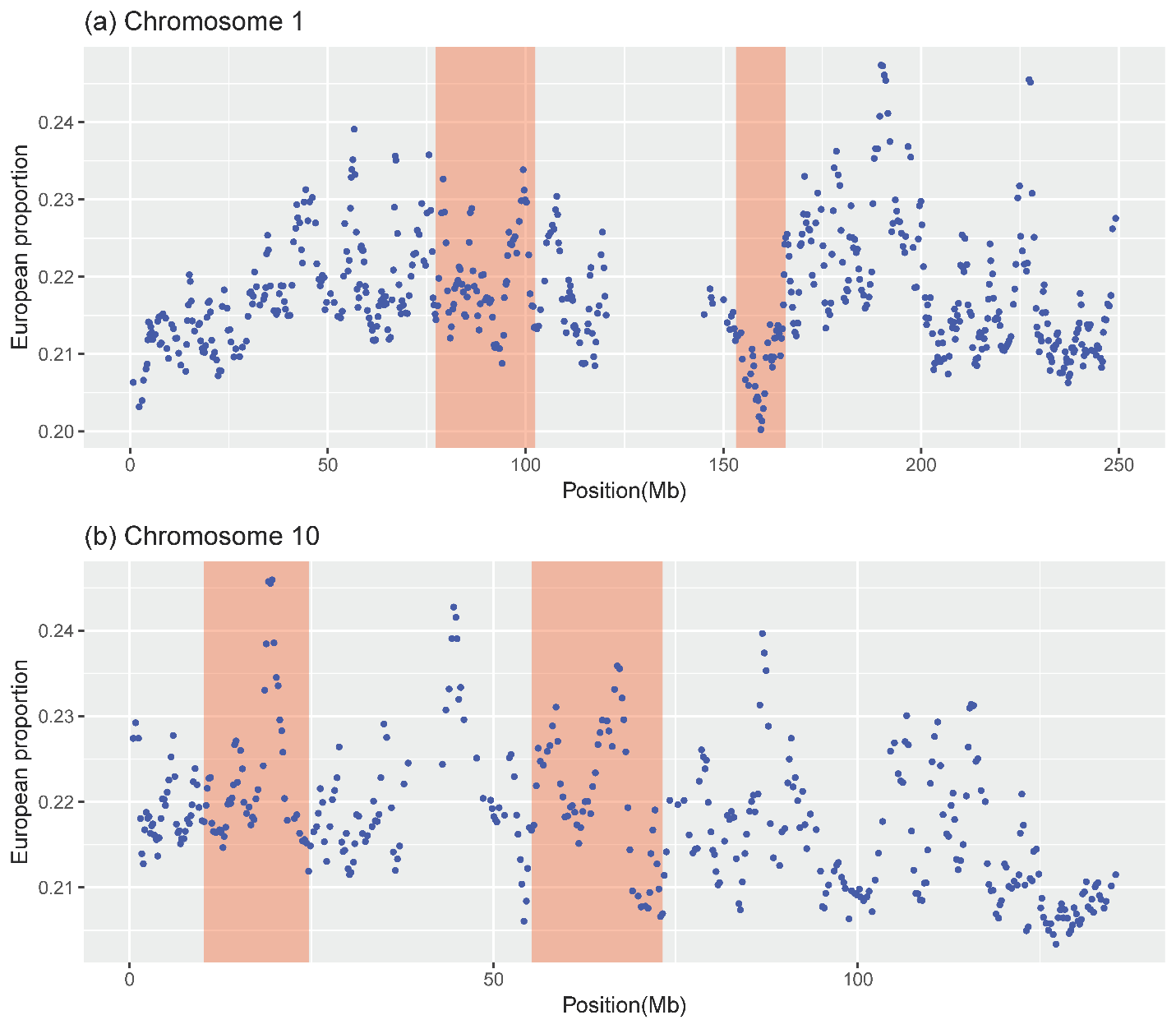


**Figure S2. Proportion of local ancestries of (a) chromosome 1 and (b) chromosome 10.** The orange shaded areas represent the epistatic regions on chromosome 1 and chromosome 10, respectively.


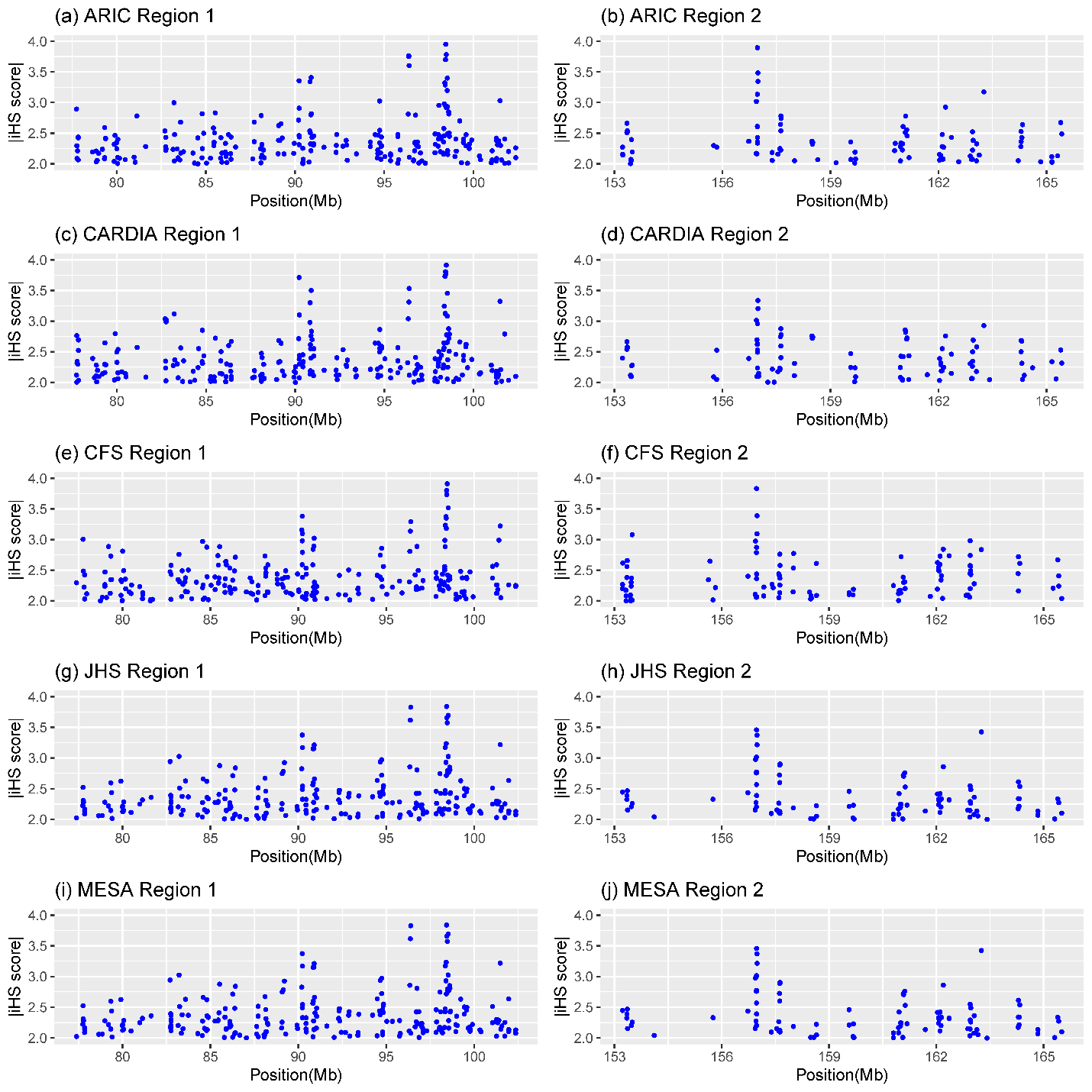


**Figure S3. The recent selection signal (|iHS| > 2) on the epistatic regions on chromosome 1 in CARe cohort.** (a) and (b) are the selection signals in region 1 and region 2 in ARIC (sample size: 2485), respectively. (c) and (d) are the selection signals in region 1 and region 2 in CARDIA (sample size: 814), respectively. (e) and (f) are the selection signals in region 1 and region 2 in CFS (sample size: 137), respectively. (g) and (h) are the selection signals in region 1 and region 2 in JHS (sample size: 1228), respectively. (i) and (j) are the selection signals in region 1 and region 2 in MESA (sample size: 1574), respectively.


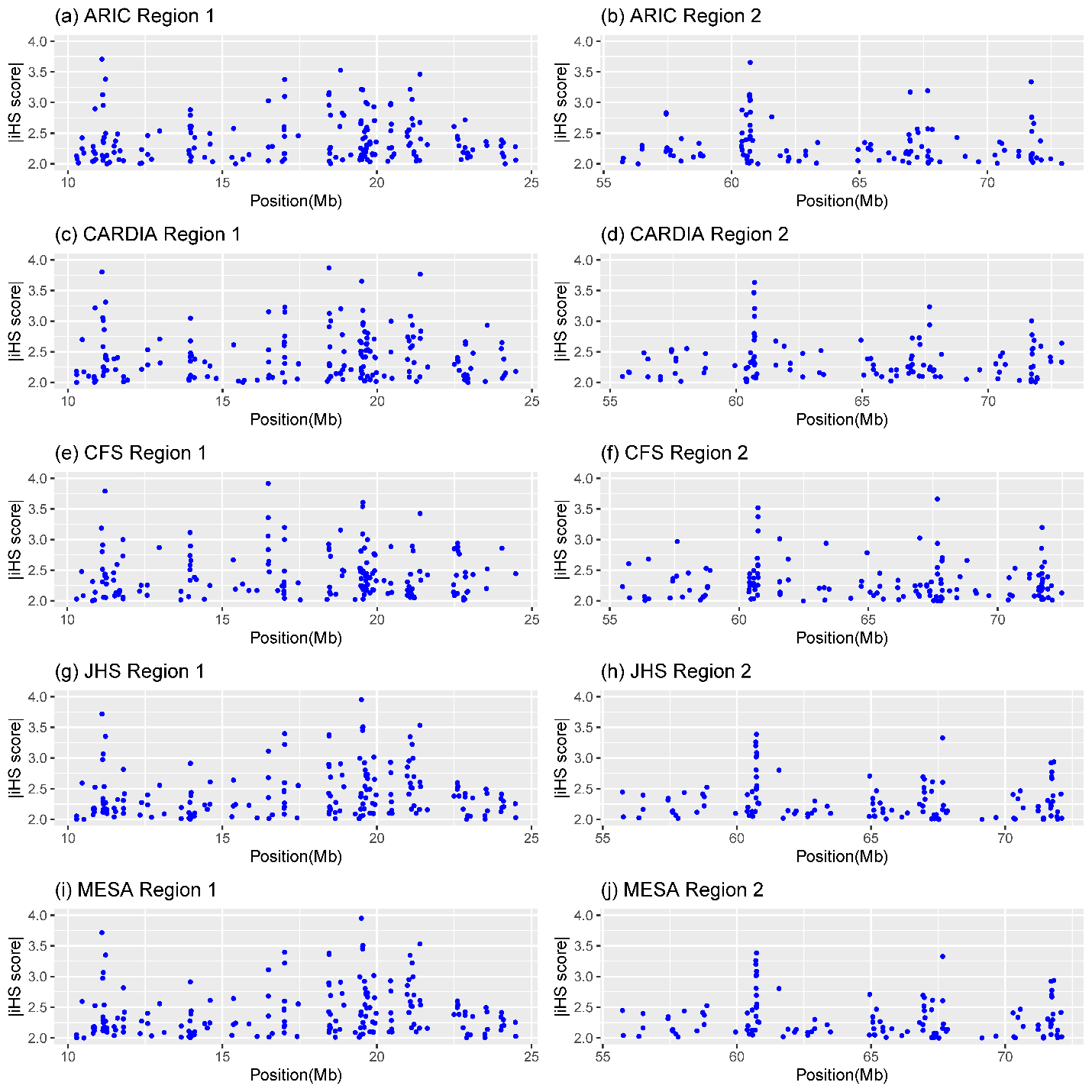


**Figure S4.** **The recent selection signal (|iHS| > 2) on the epistatic regions on chromosome 10 in CARe cohort.** (a) and (b) are the selection signals in region 1 and region 2 in ARIC (sample size: 2485), respectively. (c) and (d) are the selection signals in region 1 and region 2 in CARDIA (sample size: 814), respectively. (e) and (f) are the selection signals in region 1 and region 2 in CFS (sample size: 137), respectively. (g) and (h) are the selection signals in region 1 and region 2 in JHS (sample size: 1228), respectively. (i) and (j) are the selection signals in region 1 and region 2 in MESA (sample size: 1574), respectively.


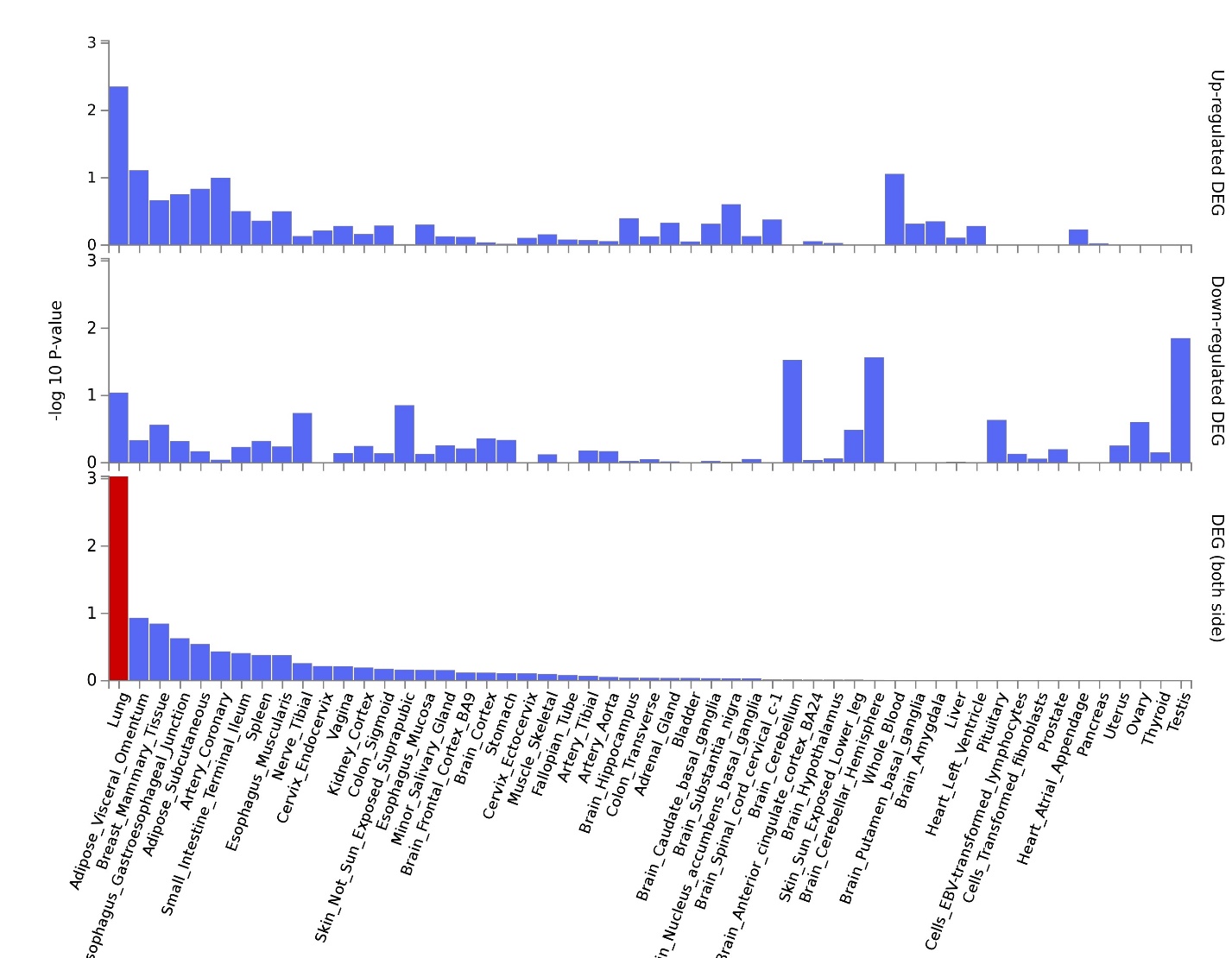


**Figure S5. The tissue expression results of co-expressed genes on 53 tissue types by GTEx in FUMA.** Significantly enriched differentially expressed genes (DEG) sets (P_bon_ < 0.05/53) are highlighted in red.


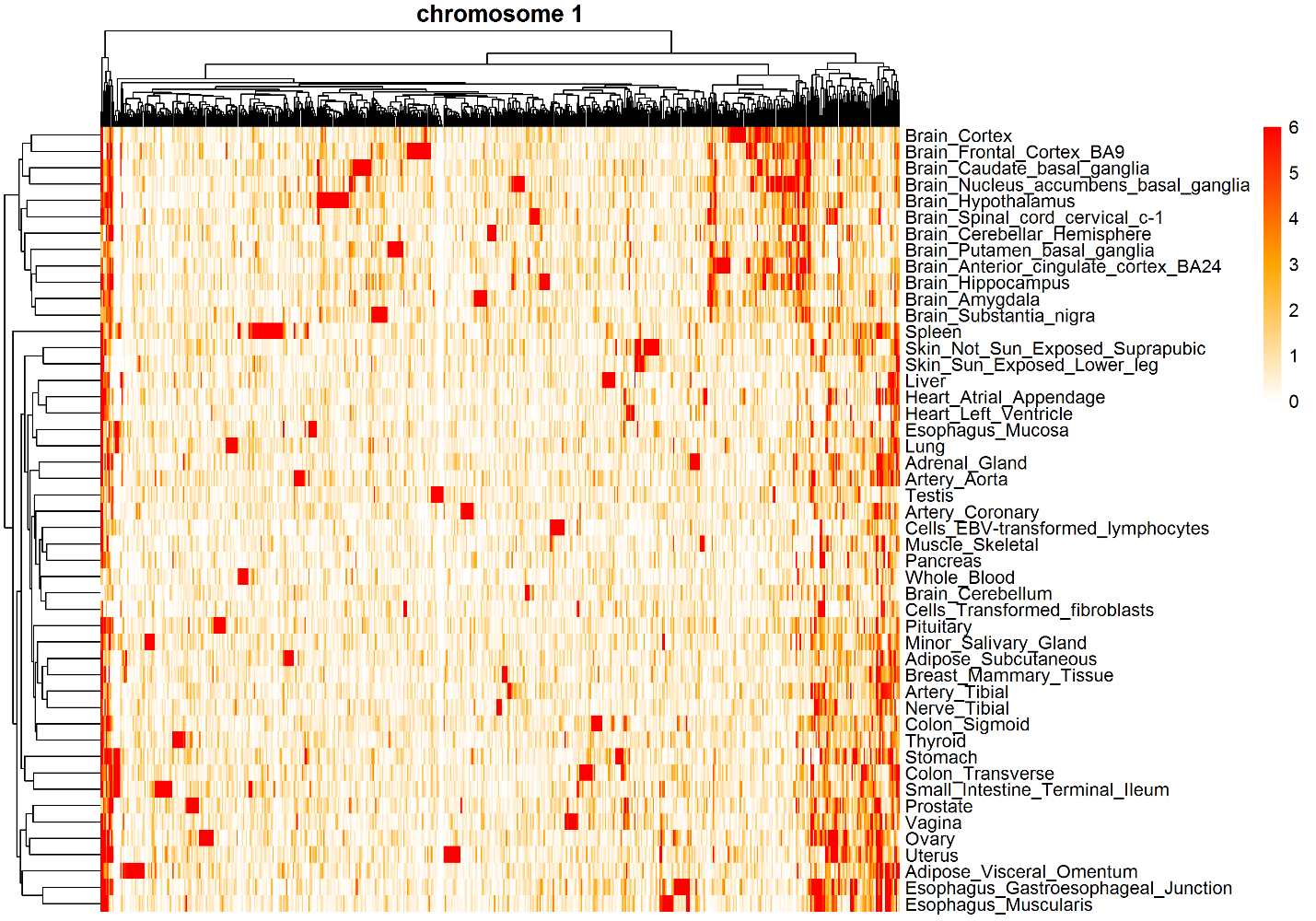


**Figure S6.** **Heatmap of P-value of significantly co-expressed gene pairs located on epistatic regions on chromosome 1 in different tissues.** Y-axis represents the names of different tissues. X-axis represents the gene pairs. These gene pairs are significantly co-expressed at least in one tissue. Red block represents the significant signals.


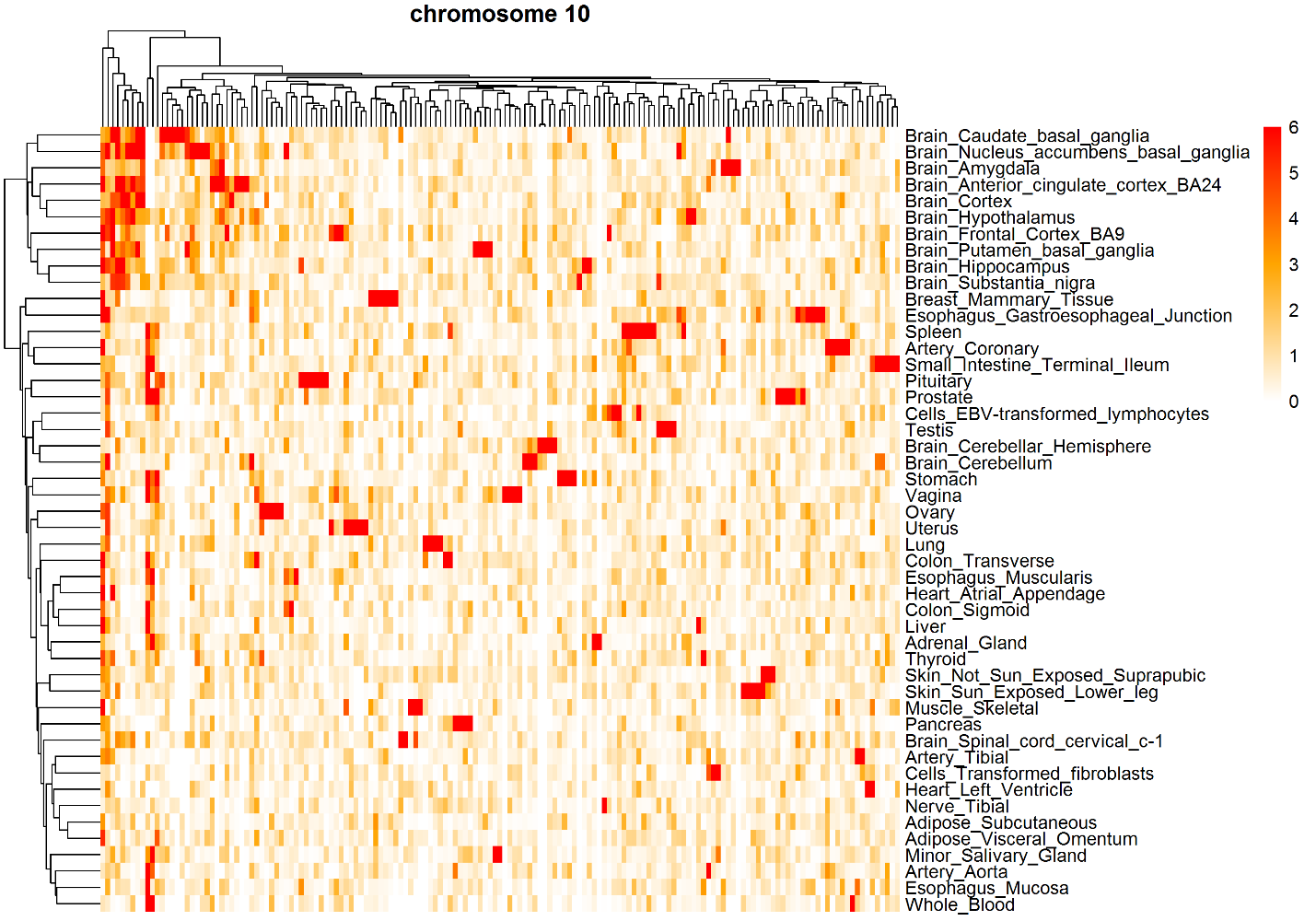


**Figure S7. Heatmap of P-value of significantly co-expressed gene pairs located on epistatic regions on chromosome 10 in different tissues.** Y-axis represents the names of different tissues. X-axis represents the gene pairs. These gene pairs are significantly co-expressed at least in one tissue. Red block represents the significant signals.


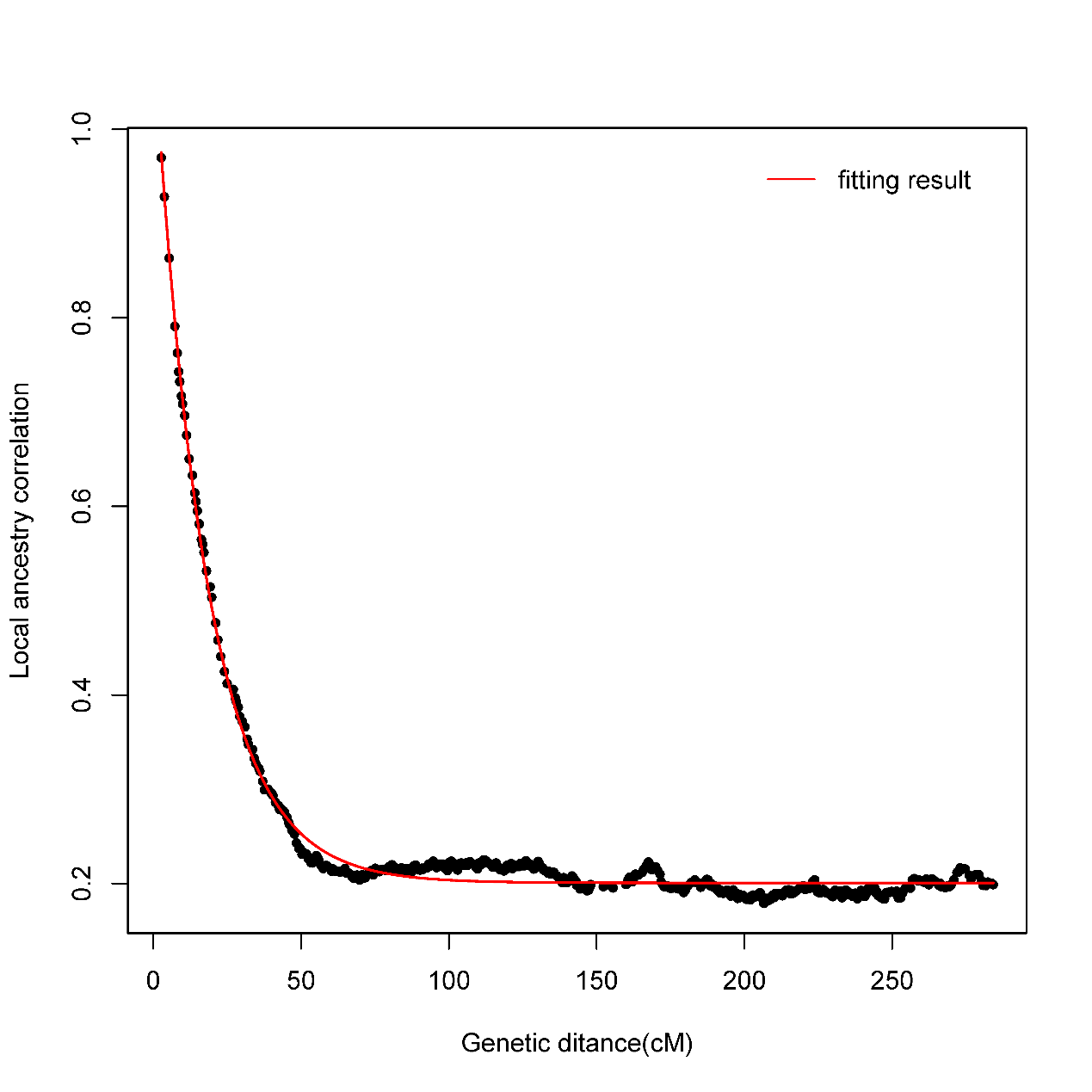


**Figure S8. An example of admixture LD decay with genetic distance.** We calculated the local ancestry correlations in the Women’s Health Initiative (WHI) cohorts, with 8,150 African Americans. Red line is the fitting results by using an exponential function.

**Supplementary Tables**

**Table S1. Correlations of Z score among CARe, FBPP and WHI cohorts.**

|  | CARe | FBPP | WHI |
| --- | --- | --- | --- |
| CARe | 1.000 | 0.241 | 0.411 |
| FBPP | 0.241 | 1.000 | 0.243 |
| WHI | 0.411 | 0.243 | 1.000 |

**Table S2. Number of crossover events between African and European chromosomes.**

| Genome region | ANCAEC^*^ | Physical length (Mb) | Genetic length (cM) | ANCAEC per Mb | ANCAEC per cM |
| --- | --- | --- | --- | --- | --- |
| Genomes between epistasis regions on chromosome 1 | 0.9078 | 50.80 | 32.88 | 0.0179 | 0.0276 |
| Genomes between epistasis regions on chromosome 10 | 1.0926 | 30.60 | 32.53 | 0.0357 | 0.0336 |
| Rest of genomes | 130.5588 | 2688.96 | 3441.05 | 0.0486 | 0.0379 |

^*^ The average number of crossovers between African and European chromosomes among 16,252 subjects.

**Table S3. Comparison of co-expressed gene pairs between the epistatic regions and other regions not overlapped with the epistatic regions on chromosome 1 and chromosome 10.**

| Region pair^*^ | Region 1 (cM)^**^ | Region 2 (cM) | Number of gene pair^***^ | Number of co-expressed gene pairs | Proportion of co-expressed gene pairs | |
| --- | --- | --- | --- | --- | --- | --- |
| Chr1: Region pair 1 | 30.88-55.66 | 88.54-105.93 | 980396 | 357 | 0.000364 | |
| Chr1: Region pair 2 | 55.88-80.66 | 113.54-130.93 | 2391635 | 775 | 0.000324 | |
| Chr1: Region pair 3 | 80.88-105.66 | 138.54-155.93 | 918181 | 224 | 0.000244 | |
| Chr1: Region pair 4 | 130.88-155.66 | 188.54-205.93 | 1423815 | 503 | 0.000353 | |
| Chr1: Region pair 5 | 180.88-205.66 | 238.54-255.93 | 1284491 | 367 | 0.000286 | |
| Chr1: Epistatic regions | 105.88-130.66 | 163.54-180.93 | 2073452 | 1049 | 0.000506 | |
| Chr10: Region pair 1 | 47.62-71.12 | 103.65-122.01 | 898579 | 268 | 0.000298 |  |
| Chr10: Region pair 2 | 97.62-121.12 | 153.65-172.01 | 389275 | 214 | 0.00055 |  |
| Chr10: Epistatic regions | 22.62-46.12 | 78.65-97.01 | 356797 | 210 | 0.000589 |  |

^*^ Region pairs were generated by moving the epistatic regions without overlap on chromosome 1.

^**^ Regions are measured in genetic distance.

^***^ Number of gene pair is the sum of gene pairs in all tissues.

**Table S4. Number of** **GWAS hits on the epistatic regions.**

| Chromosome | Region | Start (Mb) | End (Mb) | Length (Mb) | Hits number | Average number (/Mb) |
| --- | --- | --- | --- | --- | --- | --- |
| Chr 1 | Region 1 | 77.32 | 102.43 | 25.11 | 820 | 32.66 |
| Chr 1 | Region 2 | 153.22 | 165.73 | 12.51 | 844 | 67.47 |
| Chr 1 | Whole chromosome 1 | 0 | 249.25 | 249.25 | 9571 | 38.40 |
| Chr 10 | Region 1 | 10.26 | 24.59 | 14.33 | 461 | 32.17 |
| Chr 10 | Region 2 | 55.20 | 73.20 | 18.00 | 740 | 41.11 |
| Chr 10 | Whole chromosome 10 | 0 | 135.53 | 135.53 | 4930 | 36.38 |
